# Comment on Katsarelias, D., et al. “The Effect of Beta-Adrenergic Blocking Agents in Cutaneous Melanoma—A Nation-Wide Swedish Population-Based Retrospective Register Study” *Cancers* 2020, *12*, 3228

**DOI:** 10.3390/cancers13010095

**Published:** 2020-12-30

**Authors:** Vincenzo de Giorgi, Luciana Trane, Federico Venturi, Flavia Silvestri, Federica Scarfì, Sara Gandini

**Affiliations:** 1 Department of Dermatology, University of Florence, 50132 Florence, Italy; luciana.trane@gmail.com (L.T.); federico.venturi@unifi.it (F.V.); flavia.silvestri25@gmail.com (F.S.); scarfif@gmail.com (F.S.); 2 Department of Experimental Oncology, IEO European Institute of Oncology IRCSS, 20121 Milan, Italy; sara.gandini@ieo.it

We read the paper by Katsarelias et al. with great interest, regarding the effect of beta-adrenergic blocking agents on cutaneous melanoma [1]. However, the study presents some methodology biases that do not allow us to support the authors’ conclusions. The paper suffers from the unification and evaluation of multiple registries, which do not provide essential data for any of the targets for research. Unlike studies in the literature [2,3,4], thin melanomas, namely <0.75 mm, were included in the study, which are known to be low risk for progression and may render the case series unsuitable for their objectives, with the events less statistically significant by diluting the results.

Yet, the most crucial bias appears to be the inclusion criteria. The authors clarify that only patients with melanoma, who started the beta blocker after diagnosis, were considered; however, in the Materials and Methods section, it is unclear how many were excluded, as they were already in therapy prior to diagnosis. The authors selected 12,738 patients with invasive melanoma for the study; these patients crossed the Swedish Prescribed Drug Registry (SPDR), since all of them were used in the study. We question if no one had used the beta blocker before diagnosis, or if the authors selected subjects using a different approach? In addition, how could patients be considered for the beta blocker before diagnosis, and then use another drug? Furthermore, the average period of beta-blocker intake in selected patients was not assessed in the final analysis, or how long after diagnosis the therapy was started. In our opinion, based on what is known from the literature [2,3,4,5], all data are fundamental to draw statistically significant conclusions.
